# Impact of Malnutrition on the Length of Stay for Hospitalized Chimeric Antigen Receptor T-cell (CAR-T) Therapy Patients in the United States (2020)

**DOI:** 10.7759/cureus.72400

**Published:** 2024-10-25

**Authors:** Tong Ren, Alan Kerr, Olu Oyesanmi, Salman Muddassir

**Affiliations:** 1 Internal Medicine, University of South Florida (USF) Morsani College of Medicine/HCA Florida Oak Hill Hospital, Brooksville, USA; 2 Hematology and Medical Oncology, University of South Florida (USF) Morsani College of Medicine, Tampa, USA; 3 Hematology and Medical Oncology, Tampa General Hospital Cancer Institute, Tampa, USA

**Keywords:** cancer cachexia, chimeric antigen receptor (car) t-cell therapy, length of hospital stay (los), malnutrition, national inpatient sample (nis)

## Abstract

Background

Chimeric antigen receptor T-cell (CAR-T) therapy offers a promising treatment for certain malignancies but can be associated with complications. Malnutrition and cachexia are common in cancer patients and may worsen outcomes. This study investigated the impact of malnutrition on the length of hospital stay (LOS) in patients with hematologic malignancies undergoing CAR-T therapy. The analysis focused on different subpopulations, including those with acute lymphoblastic leukemia (ALL), multiple myeloma (MM), diffuse large B-cell lymphoma (DLBCL), and non-Hodgkin lymphoma (NHL) excluding DLBCL.

Methods

Utilizing the 2020 National Inpatient Sample (NIS) data, we performed survey-based mean estimation analyses for LOS across various subpopulations of CAR-T therapy patients. These subpopulations were defined by specific diagnoses: ALL, myeloma, DLBCL, and NHL excluding DLBCL. We compared the LOS between patients with and without malnutrition using STATA accounting for the complex survey design. Cachexia was included as disease-induced malnutrition.

Results

The total CAR-T population used for analyses included 439 patients, and malnutrition was present in 50 (11.39%). The overall CAR-T population demonstrated a significantly longer LOS for patients with malnutrition (30.92 days, 95% CI: 24.30 to 37.54) compared to those without malnutrition (17.97 days, 95% CI: 15.48 to 20.46, p = 0.0002). This trend held true across subgroups. Specifically, the ALL population had a significantly longer LOS with malnutrition (45.25 days, 95% CI: 35.46 to 55.04) compared to non-malnourished patients (27.58 days, 95% CI: 16.74 to 38.42, p = 0.0279). For the DLBCL population, the mean LOS was 24.47 days (95% CI: 19.22 to 29.71) with malnutrition and 17.17 days (95% CI: 13.29 to 21.04, p = 0.0161) without malnutrition. The NHL population excluding DLBCL exhibited a mean LOS of 33.86 days (95% CI: 22.66 to 45.07) for malnourished patients and 17.44 days (95% CI: 14.76 to 20.11, p = 0.0055) for non-malnourished patients. The myeloma population showed a similar trend although not statistically significant, with a mean LOS of 39.00 days (95% CI: -3.54 to 81.54) for malnourished patients and 18.03 days (95% CI: 15.02 to 21.03, p = 0.3337) for non-malnourished patients. These findings highlight significant variations in LOS across different CAR-T-treated cancer subtypes, emphasizing the impact of malnutrition on healthcare resource utilization in oncology.

Conclusion

Malnutrition is associated with a significantly longer hospital stay among patients undergoing CAR-T therapy. This trend is consistent across various subpopulations, including those with ALL, DLBCL, and NHL (excluding DLBCL). While the impact of malnutrition on LOS was not statistically significant in the myeloma population, this could potentially be attributed to the smaller sample size in this group. Overall, these findings underscore the critical role of nutritional status in managing patients undergoing CAR-T therapy. Future studies should investigate the most effective methods for identifying and treating malnutrition in this patient population to reduce hospital stays and optimize overall patient care.

## Introduction

Chimeric antigen receptor T-cell (CAR-T) therapy has emerged as a groundbreaking treatment for patients with refractory or relapsed B-cell malignancies [1-4]. By engineering a patient's T-cells to target specific cancer cells, CAR-T therapy has shown remarkable efficacy, particularly with the use of CD19-CAR-T cells in B cell malignancies [5,6]. However, despite its revolutionary potential, CAR-T therapy is associated with significant adverse outcomes, including multiple organ dysfunction, sepsis, disseminated intravascular coagulation (DIC), cytokine release syndrome (CRS), and immune effector cell-associated neurotoxicity syndrome (ICANS) [7,8].

Malnutrition and cachexia are prevalent in cancer patients, often exacerbated by treatment-related factors such as anorexia, nausea, and fatigue [9-12]. CAR-T therapy, with its associated toxicities such as CRS and ICANS, may increase the risk of these conditions [13-15]. Patients with malnutrition or cachexia often experience poorer outcomes, including increased infection risk and prolonged hospital stays [16]. Given the potential impact of nutritional status on CAR-T therapy efficacy and patient survival, this study investigates the relationship between malnutrition and length of hospital stay (LOS) in this patient population.

CRS, a potentially life-threatening side effect, occurs in a significant proportion of CAR-T patients, leading to severe inflammatory responses. Similarly, ICANS affects a substantial number of patients and can range from mild (e.g., fatigue, headache, and tremor) to severe symptoms (e.g., seizures and coma). These side effects, combined with the intensive care unit (ICU) admissions often required for CAR-T therapy patients, further exacerbate the risk of malnutrition and cachexia [17]. ICU stays are commonly associated with muscle wasting and increased metabolic demands, both of which can severely impact nutritional status.

The relationship between malnutrition, cachexia, and CAR-T therapy outcomes underscores the need for comprehensive nutritional assessment and intervention in this patient population. Recent studies have shown that pre-treatment malnutrition and cachexia are significantly associated with poorer survival rates and increased complications such as sepsis and prolonged hospital stays [18-21]. These findings highlight the critical importance of early nutritional screening and tailored dietary interventions to optimize treatment outcomes for patients receiving CAR-T therapy [22].

In this study, we aim to explore the impact of nutritional status on the outcomes of patients receiving CAR-T therapy using the 2020 National Inpatient Sample (NIS) data. By examining the incidence and consequences of malnutrition and cachexia in this context, we seek to identify potential pathways for improving patient care and therapeutic efficacy in this novel treatment landscape.

## Materials and methods

Data source

This retrospective cohort study utilized the 2020 NIS database, which is part of the Healthcare Cost and Utilization Project (HCUP) sponsored by the Agency for Healthcare Research and Quality (AHRQ). The NIS database is the largest publicly available all-payer inpatient healthcare database in the United States, providing a representative sample of hospital discharges across the nation. The NIS contains data on hospital inpatient stays, including patient demographics, diagnoses, procedures, LOS, and discharge status. The database is designed to represent a stratified sample of approximately 20% of US community hospitals, enabling national estimates.

Study population

The study population consisted of adult patients (aged 18 years and older) hospitalized in the United States in 2020 who underwent CAR-T therapy. Patients were stratified into two groups based on the presence or absence of malnutrition, including cachexia. Malnutrition was defined as a state of undernutrition characterized by insufficient intake of protein and energy, leading to loss of fat and muscle mass and resulting in diminished physical function and immune response. Since the NIS database does not provide BMI data, malnutrition was identified using ICD-10-CM codes for malnutrition and cachexia (E40-E46, R64). This method of identifying malnutrition has been used in other large administrative database studies [23]. In addition to malnutrition, the study included patients diagnosed with specific hematologic malignancies commonly treated with CAR-T therapy, such as acute lymphoblastic leukemia (ALL), multiple myeloma (MM), diffuse large B-cell lymphoma (DLBCL), and other non-Hodgkin lymphomas (oNHL). The incidence of sepsis, a severe and potentially life-threatening condition, was also considered and identified using a broad range of ICD-10-CM codes. A complete list of ICD codes used in this study can be found in the appendix.

The study aimed to examine the impact of malnutrition on hospital outcomes, particularly among patients undergoing CAR-T therapy for these hematologic malignancies, focusing on in-hospital mortality, LOS, incidence of sepsis, and total hospital charges.

Variables

This study focused on several key variables related to the demographic and clinical characteristics of patients undergoing CAR-T therapy, as well as hospital outcomes. Patient demographics, including age, gender, race/ethnicity, and median household income quartile, were analyzed to understand the population characteristics. Clinical variables included the Charlson Comorbidity Index (CCI), which was calculated to quantify the burden of comorbid conditions, with patients categorized by scores of 0, 1, 2, or 3+. Insurance status was categorized included Medicare, Medicaid, private insurance, self-pay, no charge, and other forms of insurance. The type of admission was classified as either elective or non-elective, and hospitals were categorized by geographic location according to U.S. Census divisions. Hospital outcomes analyzed in this study included in-hospital mortality, defined as death occurring during hospitalization, and LOS, which was measured in days. Total hospital charges were calculated as the total charges incurred during the hospitalization. Sepsis was identified using a broad range of ICD-10-CM codes. These variables were analyzed to assess the impact of malnutrition, specific hematologic malignancies, and sepsis on hospital outcomes among patients undergoing CAR-T therapy. These variables were analyzed to assess the impact of malnutrition, specific hematologic malignancies, and sepsis on hospital outcomes among patients undergoing CAR-T therapy.

Statistical analysis

Descriptive statistics were used to compare baseline characteristics between malnourished and non-malnourished patients. Continuous variables were expressed as means and standard deviations, while categorical variables were presented as frequencies and percentages. The design-adjusted chi-square test (F-test equivalent)) and survey-adjusted linear regression (t-test equivalent) were used for categorical and continuous variables, respectively. Multivariable logistic regression was performed to adjust for potential confounders, including age, gender, race, CCI scores, and hospital characteristics. A p-value of <0.05 was considered statistically significant. Data analyses were conducted using Stata Statistical Software: Release 16 (StataCorp., College Station, TX).

## Results

The study included 439 CAR-T therapy hospitalizations in 2020, with 11.39% of patients (n = 50) identified as malnourished and 389 without malnutrition (Table 1). 

**Table 1:** Overall CAR-T therapy patient demographics and hospital characteristics

Characteristics	CAR-T patients with malnutrition	CAR-T patients without malnutrition	Total CAR-T therapy hospitalizations	P-value	Test used	Test statistics
Number of hospitalizations	50	389	439			
Mean age (years)	56.7	57.4	57.3	0.822	Survey-adjusted linear regression (t test equivalent)	F(1, 1068) = 0.05, t = -0.23
Gender				0.32	Design-adjusted Chi-square test (F-test equivalent)	F(1, 1068) = 0.9976
Male	34 (68%)	232 (59.64%)	266 (60.59%)			
Female	16 (32%)	157 (40.36%)	173 (39.41%)			
Race	48	378	426	0.0247	Design-adjusted Chi-square test (F-test equivalent)	F(4.53, 4841.24) = 2.6685
White	32 (66.67%)	292 (77.25%)	325 (76.29%)			
Black	6 (12.50%)	26 (6.88%)	32 (7.51%)			
Hispanic	7 (14.58%)	36 (9.52%)	43 (10.09%)			
Asian or Pacific Islander	1 (2.08%)	12 (3.17%)	13 (3.05%)			
Native American	1 (2.08%)	0 (0%)	1 (0.23%)			
Other	1 (2.08%)	12 (3.17%)	13 (3.05%)			
Median household income	48	374	422	0.4738	Design-adjusted Chi-square test (F-test equivalent)	F(2.96, 3156.09) = 0.8335
0-25th percentile	11 (22.92%)	59 (15.78%)	70 (16.59%)			
26th to 50th percentile	9 (18.75%)	76 (20.32%)	85 (20.14%)			
51st to 75th percentile	13 (27.08%)	97 (25.93%)	110 (26.07%)			
76th to 100th percentile	15 (31.25%)	142 (37.97%)	157 (37.20%)			
Insurance status	49	389	438	0.7993	Design-adjusted Chi-square test (F-test equivalent)	F(4.10, 4383.50) = 0.4199
Medicare	22 (44.90%)	148 (38.05%)	170 (38.81%)			
Medicaid	5 (10.20%)	33 (8.48%)	38 (8.68%)			
Private insurance	20 (40.82%)	187 (48.07%)	207 (47.26%)			
Self-pay	0 (0%)	6 (1.54%)	6 (1.37%)			
No charge	0 (0%)	2 (0.51%)	2 (0.46%)			
Other	2 (4.08%)	13 (3.34%)	15 (3.42%)			
Charlson Comorbidity Index Score				0.6901	Design-adjusted Chi-square test (F-test equivalent)	F(2.88, 3080.61) = 0.4780
0	0 (0%)	1 (0.26%)	1 (0.23%)			
1	0 (0%)	1 (0.26%)	1 (0.23%)			
2	25 (50%)	226 (58.10%)	251 (57.18%)			
3 or more	25 (50%)	161 (41.39%)	186 (42.37%)			
Elective versus non-elective admission				0.737	Design-adjusted Chi-square test (F-test equivalent)	F(1, 1068) = 0.1130
Non-elective	15 (31.25%)	106 (27.25%)	121 (27.56%)			
Elective	35 (70%)	283 (72.75%)	318 (72.44%)			
Census division of the hospital				0.6958	Design-adjusted Chi-square test (F-test equivalent)	F(5.33, 5690.83) = 0.6190
New England	2 (4.00%)	41 (10.54%)	43 (9.79%)			
Middle Atlantic	8 (16.00%)	69 (17.74%)	77 (17.54%)			
East North Central	10 (20.00%)	62 (15.94%)	72 (16.40%)			
West North Central	5 (10.00%)	31 (7.97%)	36 (8.20%)			
South Atlantic	8 (16.00%)	62 (15.94%)	70 (15.95%)			
East South Central	0 (0%)	11 (2.83%)	11 (2.51%)			
West South Central	5 (10.00%)	30 (7.71%)	35 (7.97%)			
Mountain	0 (0%)	11 (2.83%)	11 (2.51%)			
Pacific	12 (24.00%)	72 (18.51%)	84 (19.13%)			
Hospital bed size				0.7433	Design-adjusted Chi-square test (F-test equivalent)	F(1.85, 1974.11) = 0.2735
Small	7 (14.00%)	55 (14.14%)	62 (14.12%)			
Medium	9 (18.00%)	58 (14.91%)	67 (15.26%)			
Large	34 (68.00%)	276 (70.95%)	310 (70.61%)			
Mortality rate (%)	7 (14%)	9 (2.31%)	16 (3.64%)	0	Design-adjusted Chi-square test (F-test equivalent)	F(1, 1068) = 16.6785
Sepsis	10 (20%)	25 (6.43%)	35 (7.97%)	0.0015	Design-adjusted Chi-square test (F-test equivalent)	F(1, 1068) = 10.1148
Mean hospital charges	1500193	949102.9	1012887	0.0018	Survey-adjusted linear regression (t test equivalent)	F(1, 1058) = 9.77

Patient characteristics, including age, gender, income, insurance status, and hospital size, did not show statistically significant differences between malnourished and non-malnourished groups, which helps minimize potential confounding variables in assessing LOS and mortality outcomes.

**Figure 1:**
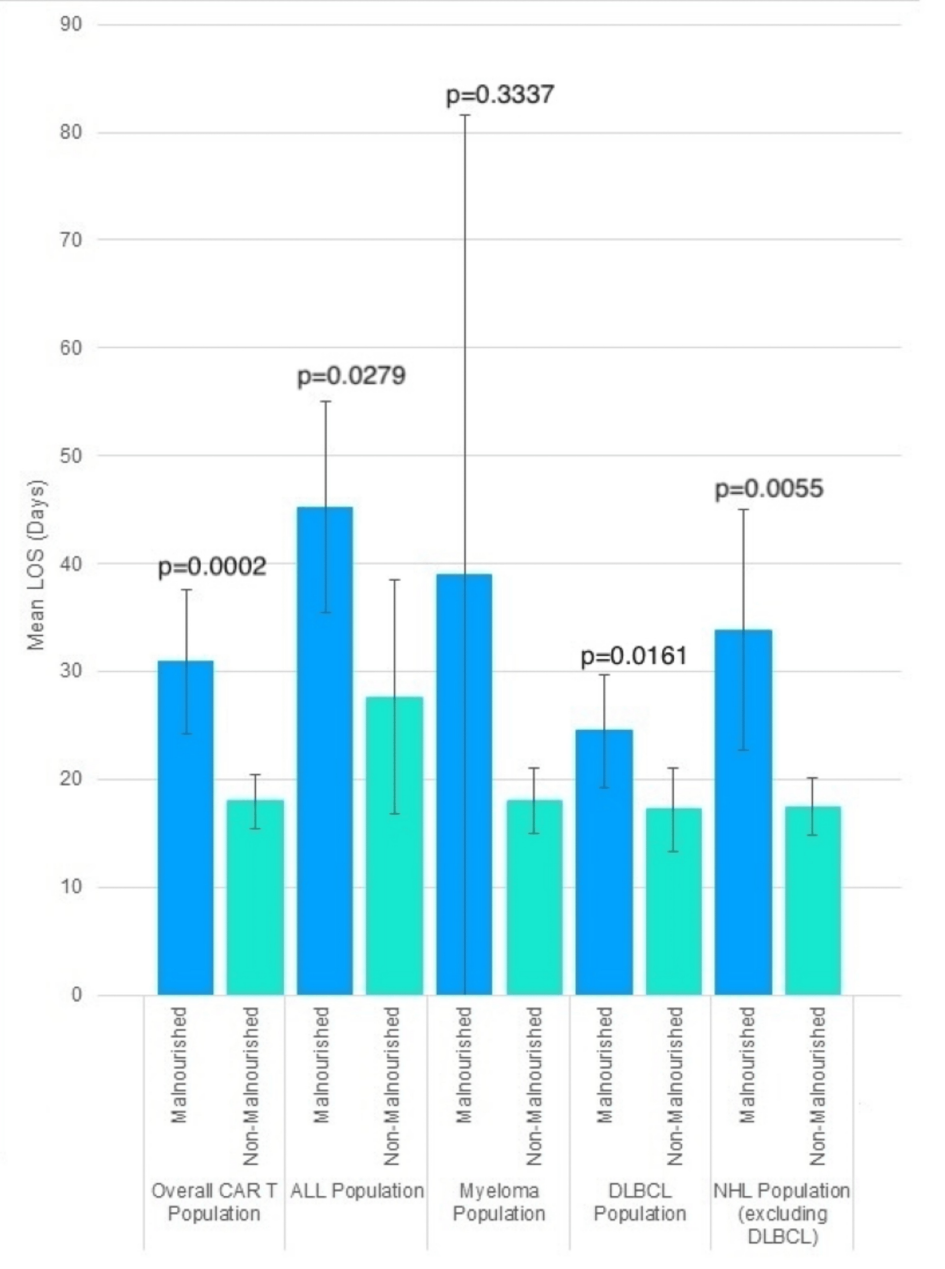
Mean length of stay (LOS) for malnourished vs. non-malnourished patients undergoing CAR T-cell therapy

Malnourished patients had a significantly longer mean LOS compared to non-malnourished patients, with a mean LOS of 30.92 days versus 17.97 days (p = 0.0002). This trend was observed across multiple subpopulations. For instance, in the ALL group, malnourished patients had a mean LOS of 45.25 days compared to 27.58 days for non-malnourished patients (p = 0.0279). Similarly, in patients with DLBCL, malnourished patients had a mean LOS of 24.47 days versus 17.17 days for non-malnourished patients (p = 0.0161). In the oNHL group, malnourished patients had a significantly longer mean LOS of 33.86 days compared to 17.44 days for non-malnourished patients (p = 0.0055). In the MM subgroup, the difference in LOS, although not statistically significant, showed a trend toward longer stays in malnourished patients (39.00 days vs. 18.03 days, p = 0.3337) (Figure 1).

Malnutrition was also associated with significantly higher in-hospital mortality and sepsis rates. The overall in-hospital mortality rate for malnourished patients was 14% compared to 2.31% for non-malnourished patients (p = 0.0004). Sepsis occurred in 20% of malnourished patients, compared to 6.43% of non-malnourished patients (p = 0.0027). Subgroup analyses revealed notable findings: in the ALL subgroup, the mortality rate for malnourished patients was 50%, compared to 2.63% for non-malnourished patients (p = 0.0077), and sepsis occurred in 75% of malnourished ALL patients compared to 13.16% of non-malnourished patients (p = 0.028) (Table 2).

**Table 2:** ALL CAR-T therapy patient demographics and hospital characteristics ALL: acute lymphoblastic leukemia, CAR-T: chimeric antigen receptor T-cell

Characteristics	CAR-T patients with malnutrition	CAR-T patients without malnutrition	Total CAR-T therapy hospitalizations	P-value	Test used	Test statistic
Number of hospitalizations	4	38	42			
Mean age (years)	16.75	20.37	20.02	0.5127	Survey-adjusted linear regression (t-test equivalent)	F(1, 707) = 0.43
Gender				0.4131	Design-adjusted Chi-square test (F-test equivalent)	F(1, 707) = 0.6705
Male	3 (75%)	20 (52.63%)	23 (54.76%)			
Female	1 (25%)	18 (47.37%)	19 (45.24%)			
Race				Not applicable	Unable to compute due to zero cells in the table	Not applicable
White	2 (50%)	18 (47.37%)	20 (47.62%)			
Black	0 (0%)	1 (2.63%)	1 (2.38%)			
Hispanic	2 (50%)	13 (34.21%)	15 (35.71%)			
Asian or Pacific Islander	0 (0%)	1 (2.63%)	1 (2.38%)			
Native American	0 (0%)	0 (0%)	0 (0%)			
Other	0 (0%)	3 (7.89%)	3 (7.14%)			
Median household income				Not applicable	Unable to compute due to zero cells in the table	Not applicable
0 to 25th percentile	0 (0%)	6 (15.79%)	6 (14.29%)			
26th to 50th percentile	1 (25%)	8 (21.05%)	9 (21.43%)			
51st to 75th percentile	0 (0%)	8 (21.05%)	8 (19.05%)			
76th to 100th percentile	3 (75%)	14 (36.84%)	17 (40.48% )			
Insurance status				Not applicable	Unable to compute due to zero cells in the table	Not applicable
Medicare	0 (0%)	2 (5.26%)	2 (4.76%)			
Medicaid	1 (25%)	14 (36.84%)	15 (35.71%)			
Private insurance	2 (50%)	18 (47.37%)	20 (47.62%)			
Self-pay	0 (0%)	0 (0%)	0 (0%)			
No charge	0 (0%)	0 (0%)	0 (0%)			
Other	0 (0%)	4 (10.53%)	4 (9.52%)			
Charlson Comorbidity Index Score				Not applicable	Unable to compute due to zero cells in the table	Not applicable
0	0 (0%)	0 (0%)	0 (0%)			
1	0 (0%)	0 (0%)	0 (0%)			
2	4 (100%)	29 (76.32%)	33 (78.57%)			
3 or more	0 (0%)	9 (23.68%)	9 (21.43%)			
Elective versus non-elective admission				0.7141	Design-adjusted Chi-square test (F-test equivalent)	F(1, 707) = 0.1343
Non-elective	1 (25%)	13 (34.21%)	14 (33.33%)			
Elective	3 (75%)	25 (65.79%)	28 (66.67%)			
Census division of hospital				0.6634	Design-adjusted Chi-square test (F-test equivalent)	F(5.27, 3726.36) = 0.6582
New England	0 (0%)	3 (7.89%)	3 (7.14%)			
Middle Atlantic	0 (0%)	7 (18.42%)	7 (16.67%)			
East North Central	1 (25%)	6 (15.79%)	7 (16.67%)			
West North Central	0 (0%)	2 (5.26%)	2 (4.76%)			
South Atlantic	0 (0%)	8 (21.05%)	8 (19.05%)			
East South Central	0 (0%)	3 (7.89%)	3 (7.14%)			
West South Central	0 (0%)	1 (2.63%)	1 (2.38%)			
Mountain	0 (0%)	0 (0%)	0 (0%)			
Pacific	3 (75%)	8 (21.05%)	11 (26.19%)			
Hospital bed size				0.3269	Design-adjusted Chi-square test (F-test equivalent)	F(1.84, 1298.80) = 1.1078
Small	0 (0%)	8 (21.05%)	8 (19.05%)			
Medium	2 (50%)	9 (23.68%)	11 (26.19%)			
Large	2 (50%)	21 (55.26%)	23 (54.76%)			
Mortality rate (%)	2 (50%)	1 (2.63%)	3 (7.14%)	0.0002	Design-adjusted Chi-square test (F-test equivalent)	F(1, 707) = 13.6073
Sepsis	3 (75%)	5 (13.16%)	8 (19.05%)	0.0059	Design-adjusted Chi-square test (F-test equivalent)	F(1, 707) = 7.6314
Mean hospital charges (US dollars)	2814420	921517.3	1101793	0.0083	Survey-adjusted linear regression (t-test equivalent)	F(1, 707) = 7.00

In the MM subgroup, while no deaths were recorded among the malnourished patients, sepsis was more prevalent, occurring in 50% of malnourished patients compared to 0% of non-malnourished patients (p = 0.000) (Table 3).

**Table 3:** Myeloma CAR-T therapy patient demographics and hospital characteristics

Characteristics	CAR-T patients with malnutrition	CAR-T patients without malnutrition	Total CAR-T therapy hospitalizations	P-value	Test used	Test statistics
Number of hospitalizations	4	36	40			
Mean Age (years)	68	63.3	63.8	0.0105	Survey-adjusted linear regression (t-test equivalent)	F(1, 605) = 6.58
Gender				0.434	Design-adjusted Chi-square test (F-test equivalent)	F(1, 605) = 0.6128
Male	2 (50%)	24 (66.67%)	26 (65%)			
Female	2 (50%)	12 (33.33%)	14 (35%)			
Race				Not applicable	Unable to compute due to zero cells in the table	Not applicable
White	4 (100%)	27 (75%)	31 (77.5%)			
Black	0 (0%)	5 (13.89%)	5 (12.5%)			
Hispanic	0 (0%)	2 (5.56%)	2 (5%)			
Asian or Pacific Islander	0 (0%)	1 (2.78%)	1 (2.5%)			
Native American	0 (0%)	0 (0%)	0 (0%)			
Other	0 (0%)	0 (0%)	0 (0%)			
Median household income				0.3213	Design-adjusted Chi-square test (F-test equivalent)	F(1.98, 1199.95) = 1.1356
0 to 25th percentile	2 (50%)	6 (16.67%)	8 (20%)			
26th to 50th percentile	0 (0%)	7 (19.44%)	7 (17.5%)			
51st to 75th percentile	1 (25%)	9 (25%)	10 (25%)			
76th to 100th percentile	1 (25%)	14 (38.89%)	15 (37.5%)			
Insurance status				Not applicable	Unable to compute due to zero cells in the table	Not applicable
Medicare	2 (50%)	19 (52.78%)	21 (52.5%)			
Medicaid	0 (0%)	1 (2.78%)	1 (2.5%)			
Private insurance	2 (50%)	14 (38.89%)	16 (40%)			
Self-pay	0 (0%)	0 (0%)	0 (0%)			
No charge	0 (0%)	2 (5.56%)	2 (5%)			
Other	0 (0%)	0 (0%)	0 (0%)			
Charlson Comorbidity Index Score				Not applicable	Unable to compute due to zero cells in the table	Not applicable
0	0 (0%)	0 (0%)	0 (0%)			
1	0 (0%)	0 (0%)	0 (0%)			
2	1 (25%)	24 (66.67%)	25 (62.5%)			
3 or more	3 (75%)	12 (33.33%)	15 (37.5%)			
Elective versus non-elective admission				0.9071	Design-adjusted Chi-square test (F-test equivalent)	F(1, 605) = 0.0137
Non-elective	1 (25%)	8 (22.22%)	9 (22.5%)			
Elective	3 (75%)	28 (77.78%)	31 (77.5%)			
Census division of hospital				0.3517	Design-adjusted Chi-square test (F-test equivalent)	F(4.53, 2741.74) = 1.1102
New England	2 (50%)	3 (8.33%)	5 (12.5%)			
Middle Atlantic	0 (0%)	10 (27.78%)	10 (25%)			
East North Central	0 (0%)	5 (13.89%)	5 (12.5%)			
West North Central	0 (0%)	2 (5.56%)	2 (5%)			
South Atlantic	1 (25%)	8 (22.22%)	9 (22.5%)			
East South Central	0 (0%)	0 (0%)	0 (0%)			
West South Central	0 (0%)	3 (8.33%)	3 (7.5%)			
Mountain	0 (0%)	0 (0%)	0 (0%)			
Pacific	1 (25%)	5 (13.89%)	6 (15%)			
Hospital bed size				0.0544	Design-adjusted Chi-square test (F-test equivalent)	F(1.25, 753.68) = 3.4421
Small	1 (25%)	9 (25%)	10 (25%)			
Medium	1 (25%)	1 (2.78%)	2 (5%)			
Large	2 (50%)	26 (72.22%)	28 (70%)			
Mortality rate (%)	0 (0%)	1 (2.78%)	1 (2.5%)	0.7379	Design-adjusted Chi-square test (F-test equivalent)	F(1, 605) = 0.1121
Sepsis	2 (50%)	0 (0%)	2 (5%)	0.593	Design-adjusted Chi-square test (F-test equivalent)	F(1, 605) = 0.2859
Mean hospital charges (US dollars)	1248218	179227.3	286126.2	0.3015	Survey-adjusted linear regression (t-test equivalent)	F(1, 605) = 1.07

In the DLBCL subgroup, the mortality rate for malnourished patients was 20% compared to 1.19% for non-malnourished patients (p = 0.0099), while sepsis occurred in 40% of malnourished DLBCL patients compared to 4.76% of non-malnourished patients (p = 0.0448) (Table 4). 

**Table 4:** DLBCL CAR-T therapy patient demographics and hospital characteristics DLBCL: diffuse large B-cell lymphoma, CAR-T: chimeric antigen receptor T-cell

Characteristics	CAR-T patients with malnutrition	CAR-T patients without malnutrition	Total CAR-T therapy hospitalizations	P-value	Test used	Test statistics
Number of hospitalizations	15	84	99			
Mean age (years)	64.8	61.2619	61.79798	0.3325	Survey-adjusted linear regression (t-test equivalent)	F(1, 781) = 0.94
Gender				0.3604	Design-adjusted Chi-square test (F-test equivalent)	F(1, 781) = 0.8375
Male	11 (73.33%)	51 (60.71%)	62 (62.63%)			
Female	4 (26.67%)	33 (39.29%)	37 (37.37%)			
Race				0.0478	Design-adjusted Chi-square test (F-test equivalent)	F(4.92, 3840.03) = 2.2509
White	9 (60%)	72 (85.71%)	81 (81.82%)			
Black	2 (13.33%)	5 (5.95%)	7 (7.07%)			
Hispanic	1 (6.67%)	1 (1.19%)	2 (2.02%)			
Asian or Pacific Islander	1 (6.67%)	2 (2.38%)	3 (3.03%)			
Native American	1 (6.67%)	0 (0%)	1 (1.01%)			
Other	0 (0%)	3 (3.57%)	3 (3.03%)			
Median household income				0.6639	Design-adjusted Chi-square test (F-test equivalent)	F(2.92, 2262.49) = 0.5198
0 to 25th percentile	4 (26.67%)	16 (19.05%)	20 (20.20%)			
26th to 50th percentile	2 (13.33%)	15 (17.86%)	17 (17.17%)			
51st to 75th percentile	2 (13.33%)	20 (23.81%)	22 (22.22%)			
76th to 100th percentile	7 (46.67%)	30 (35.71%)	37 (37.37%)			
Insurance status				Not applicable	Unable to compute due to zero cells in the table	Not applicable
Medicare	8 (53.33%)	36 (42.86%)	44 (44.44%)			
Medicaid	1 (6.67%)	5 (5.95%)	6 (6.06%)			
Private insurance	6 (40%)	39 (46.43%)	45 (45.45%)			
Self-pay	0 (0%)	0 (0%)	0 (0%)			
No charge	0 (0%)	0 (0%)	0 (0%)			
Other	0 (0%)	4 (4.76%)	4 (4.04%)			
Charlson Comorbidity Index Score				Not applicable	Design-adjusted Chi-square test (F-test equivalent)	Not applicable
0	0 (0%)	0 (0%)	0 (0%)			
1	0 (0%)	0 (0%)	0 (0%)			
2	7 (46.67%)	55 (65.48%)	62 (62.63%)			
3 or more	8 (53.33%)	29 (34.52%)	37 (37.37%)			
Elective versus non-elective admission				0.5751	Design-adjusted Chi-square test (F-test equivalent)	F(1, 781) = 0.3145
Non-elective	5 (33.33%)	22 (26.19%)	27 (27.27%)			
Elective	10 (66.67%)	62 (73.81%)	72 (72.73%)			
Census division of hospital				0.8128	Design-adjusted Chi-square test (F-test equivalent)	F(4.70, 3669.84) = 0.4356
New England	0 (0%)	8 (9.52%)	8 (8.08%)			
Middle Atlantic	3 (20%)	15 (17.86%)	18 (18.18%)			
East North Central	3 (20%)	17 (20.24%)	20 (20.20%)			
West North Central	2 (13.33%)	8 (9.52%)	10 (10.10%)			
South Atlantic	3 (20%)	13 (15.48%)	16 (16.16%)			
East South Central	0 (0%)	6 (7.14%)	6 (6.06%)			
West South Central	1 (6.67%)	3 (3.57%)	4 (4.04%)			
Mountain	0 (0%)	5 (5.95%)	5 (5.05%)			
Pacific	3 (20%)	9 (10.71%)	12 (12.12%)			
Hospital bed size				0.3502	Design-adjusted Chi-square test (F-test equivalent)	F(1.60, 1250.11) = 1.0094
Small	0 (0%)	7 (8.33%)	7 (7.07%)			
Medium	3 (20%)	10 (11.90%)	13 (13.13%)			
Large	12 (80%)	67 (79.76%)	79 (79.80%)			
Mortality rate (%)	3 (20%)	1 (1.19%)	4 (4.04%)	0.0005	Design-adjusted Chi-square test (F-test equivalent)	F(1, 781) = 12.0505
Sepsis	6 (40%)	4 (4.76%)	10 (10.10%)	0.0313	Design-adjusted Chi-square test (F-test equivalent)	F(1, 781) = 4.6556
Mean hospital charges (US dollars)	1330260	1163602	1189374	0.3834	Survey-adjusted linear regression (t test equivalent)	F(1, 771) = 0.76

In the oNHL (excluding DLBCL) subgroup, the mortality rate for malnourished patients was 13.64% compared to 2.5% for non-malnourished patients (p = 0.0068), while sepsis occurred in 22.72% of malnourished NHL patients compared to 6% of non-malnourished patients (p = 0.0046) (Table 5).

**Table 5:** oNHL (excluding DLBCL) CAR-T therapy patient demographics and hospital characteristics oNHL: other non-Hodgkin lymphomas, DLBCL: diffuse large B-cell lymphoma, CAR-T: Chimeric antigen receptor T-cell

Characteristics	CAR-T patients with malnutrition	CAR-T patients without malnutrition	Total CAR-T therapy hospitalizations	P-value	Test used	Test statistics
Number of hospitalizations	22	200	222			
Mean age (years)	62.90914	62.945	62.94144	0.9856	Survey-adjusted linear regression (t-test equivalent)	F(1, 852) = 0.00
Gender				0.3521	Design-adjusted Chi-square test (F-test equivalent)	F(1, 852) = 0.8668
Male	15 (68.18%)	116 (58%)	131 (59.01%)			
Female	7 (31.82%)	84 (42%)	91 (40.99%)			
Race				0.0478	Design-adjusted Chi-square test (F-test equivalent)	F(4.92, 3840.03) = 2.2509
White	16 (72.73%)	148 (74%)	164 (73.87%)			
Black	3 (13.64%)	14 (7%)	17 (7.66%)			
Hispanic	2 (9.09%)	20 (10%)	22 (9.91%)			
Asian or Pacific Islander	0 (0%)	6 (3%)	6 (2.70%)			
Native American	0 (0%)	0 (0%)	0 (0%)			
Other	1 (4.55%)	5 (2.5%)	6 (2.70%)			
Median household income				0.6639	Design-adjusted Chi-square test (F-test equivalent)	F(2.92, 2262.49) = 0.5198
0 to 25th percentile	3 (13.64%)	25 (12.5%)	28 (12.61%)			
26th to 50th percentile	6 (27.27%)	36 (18%)	42 (18.92%)			
51st to 75th percentile	7 (31.82%)	49 (24.5%)	56 (25.23%)			
76th to 100th percentile	4 (18.18%)	79 (39.5%)	83 (37.39%)			
Insurance status				Not applicable	Unable to compute due to zero cells in the table	Not applicable
Medicare	10 (45.45%)	85 (42.5%)	95 (42.79%)			
Medicaid	3 (13.64%)	13 (6.5%)	16 (7.21%)			
Private insurance	9 (40.91%)	98 (49%)	107 (48.20%)			
Self-pay	0 (0%)	2 (1%)	2 (0.90%)			
No charge	0 (0%)	0 (0%)	0 (0%)			
Other	0 (0%)	2 (1%)	2 (0.90%)			
Charlson Comorbidity Index Score				Not applicable	Unable to compute due to zero cells in the table	Not applicable
0	0 (0%)	0 (0%)	0 (0%)			
1	0 (0%)	0 (0%)	0 (0%)			
2	11 (50%)	117 (58.5%)	128 (57.66%)			
3 or more	11 (50%)	83 (41.5%)	94 (42.34%)			
Elective versus non-elective admission				0.5751	Design-adjusted Chi-square test (F-test equivalent)	F(1, 781) = 0.3145
Non-elective	6 (27.27%)	55 (27.5%)	62 (27.93%)			
Elective	16 (72.73%)	145 (72.5%)	161 (72.52%)			
Census division of hospital				0.8128	Design-adjusted Chi-square test (F-test equivalent)	F(4.70, 3669.84) = 0.4356
New England	0 (0%)	29 (14.5%)	29 (13.06%)			
Middle Atlantic	4 (18.18%)	33 (16.5%)	37 (16.67%)			
East North Central	7 (31.82%)	30 (15%)	37 (16.67%)			
West North Central	3 (13.64%)	17 (8.5%)	20 (9.01%)			
South Atlantic	3 (13.64%)	28 (14%)	31 (13.96%)			
East South Central	0 (0%)	1 (0.5%)	1 (0.45%)			
West South Central	2 (9.09%)	20 (10%)	22 (9.91%)			
Mountain	0 (0%)	2 (1%)	2 (0.90%)			
Pacific	3 (13.64%)	40 (20%)	43 (19.37%)			
Hospital bed size				0.3502	Design-adjusted Chi-square test (F-test equivalent)	F(1.60, 1250.11) = 1.0094
Small	4 (18.18%)	22 (11%)	26 (11.71%)			
Medium	3 (13.64%)	30 (15%)	33 (14.86%)			
Large	15 (68.18%)	148 (74%)	163 (73.42%)			
Mortality rate (%)	3 (13.64%)	5 (2.5%)	8 (3.6%)	0.0068	Design-adjusted Chi-square test (F-test equivalent)	F(1, 852) = 7.3485
Sepsis	5 (22.72%)	12 (6%)	17 (7.66%)	0.0046	Design-adjusted Chi-square test (F-test equivalent)	F(1, 852) = 8.0683
Mean hospital charges (US dollars)	1734461	1159878	1217077	0.0764	Survey-adjusted linear regression (t-test equivalent)	F(1, 852) = 3.15

Hospital charges were also significantly higher for malnourished patients, with a mean charge of $1,500,193 compared to $949,102.9 for non-malnourished patients (p = 0.002) (Table 1). This difference emphasizes the increased healthcare resource utilization associated with malnutrition in CAR-T therapy patients.

These findings highlight the critical impact of malnutrition on hospital outcomes, particularly LOS, in-hospital mortality, and sepsis, across various hematologic malignancies. The need for larger datasets and further studies is underscored by the smaller sample sizes in certain subpopulations, such as myeloma, which may limit statistical significance despite observed trends.

## Discussion

This study sought to evaluate the impact of malnutrition on clinical outcomes among hospitalized patients undergoing CAR-T therapy for hematologic malignancies. The findings demonstrate that malnutrition is associated with worse hospital outcomes in this population, highlighting the critical need for nutritional assessment and intervention in patients undergoing CAR-T therapy.

The prevalence of malnutrition in our study was relatively low, affecting 11.39% of patients. However, its impact on outcomes was profound. Patients with malnutrition had significantly higher in-hospital mortality rates, longer hospital stays, and greater hospital charges compared to those without malnutrition. These findings align with prior studies demonstrating that malnutrition is a strong predictor of poor outcomes in various patient populations, including cancer patients [20-22,24-26]. 

Importantly, this study builds upon existing literature by focusing on CAR-T therapy recipients - a population where malnutrition has not been as extensively studied. Given the complexity and immune-compromised state of these patients, our results emphasize the heightened vulnerability of malnourished individuals undergoing this novel and intensive treatment [27]. CAR-T therapy involves significant immunologic modulation, which can exacerbate malnutrition's detrimental effects on immune function, as seen in previous studies focusing on oncology patients [20].

In addition, we observed a significantly higher incidence of sepsis among malnourished patients. Sepsis is a life-threatening condition often driven by an impaired immune response, and its heightened occurrence in malnourished CAR-T patients may be related to their already compromised immune systems [16]. This finding is consistent with prior research showing that malnutrition predisposes cancer patients to infections due to weakened immune defenses [25]. Furthermore, sepsis has been identified as a major complication in CAR-T therapy patients, particularly those with existing comorbidities, such as malnutrition [13]. Our study adds to this understanding by emphasizing the additional burden that malnutrition places on the risk of severe infections in this already at-risk population [18].

Our analysis also revealed significant differences in racial distributions between malnourished and non-malnourished patients undergoing CAR-T therapy (F(4.53, 4841.24) = 2.6685, p = 0.0247). White patients represented the largest proportion of both malnourished (66.67%) and non-malnourished (77.25%) groups, although malnutrition was more prevalent among non-White racial groups. Specifically, Black and Hispanic patients had higher proportions of malnutrition (12.50% and 14.58%, respectively) compared to their representation in the non-malnourished group (6.88% and 9.52%, respectively) (Table 1).

These disparities may reflect underlying socioeconomic factors, access to care, or nutritional support that disproportionately affect racial minorities. Previous studies have documented that Black and Hispanic cancer patients often experience poorer health outcomes due to structural inequities in healthcare access, nutritional support, and treatment availability [28,29]. Our findings suggest that racial disparities may also extend to malnutrition risk among CAR-T therapy recipients, further complicating their clinical outcomes. Given the novel and resource-intensive nature of CAR-T therapy, ensuring equitable access to nutritional interventions and support for racially diverse populations is critical. Addressing these disparities could improve outcomes for minority patients, as adequate nutrition is vital for both overall health and immune recovery, particularly in those undergoing intensive therapies like CAR-T therapy [30].

There was a significantly higher incidence of sepsis among malnourished patients. Sepsis is a life-threatening condition that can arise from an impaired immune response, which may be exacerbated by malnutrition. The high incidence of sepsis among malnourished patients underscores the importance of early recognition and aggressive management of infections in this vulnerable population. Although limited by smaller sample sizes, the subgroup analyses showed trends consistent with the primary analysis, reinforcing the association between malnutrition and worse clinical outcomes in CAR-T therapy patients.

Despite the compelling evidence of the adverse effects of malnutrition, it is important to recognize that this study has several limitations. First, the retrospective nature of the analysis limits the ability to establish causality. Second, the data were derived from a large administrative database, which may be subject to coding errors and lack detailed clinical information, such as body mass index before and after treatment, the severity of malnutrition, and specific nutritional interventions. Finally, the study population was limited to patients hospitalized in the United States in 2020, which may limit the generalizability of the findings to other settings and time periods.

In conclusion, our findings underscore the importance of comprehensive nutritional assessments and early interventions in patients undergoing CAR-T therapy. As this therapeutic approach becomes more widespread, understanding how pre-existing conditions like malnutrition affect outcomes will be essential for improving patient management and reducing treatment-related complications [11]. Integrating nutritional support strategies, as recommended in previous studies on cancer patients, maybe a critical component in improving clinical outcomes for CAR-T recipients [31]. This study also highlights the critical role of nutritional status in determining outcomes for patients undergoing CAR-T therapy. Routine nutritional assessment and early intervention could be beneficial in improving outcomes for these patients. Future studies should focus on prospective evaluations of nutritional interventions in this population to better understand their potential to improve survival and reduce healthcare costs.

## Conclusions

Our study underscores the critical influence of malnutrition on the outcomes of patients undergoing CAR-T therapy for hematologic malignancies. Malnutrition was associated with significantly higher in-hospital mortality, increased incidence of sepsis, prolonged hospital stays, and greater healthcare costs. These findings highlight the necessity for routine nutritional screening and timely interventions to optimize clinical outcomes and enhance the quality of care for this vulnerable population. Future research should focus on prospective studies to explore the efficacy of targeted nutritional support in improving survival and reducing complications in patients undergoing CAR-T therapy.

## Appendices

List of ICD codes used in this study 

**Table 6:** Diagnoses and procedure codes used in the study

Diagnosis/Procedure	ICD-10-CM/ICD-10-PCS Codes
CAR T-cell therapy	XW033C3, XW043C3
Malnutrition	E40, E41, E42, E43, E44, E45, E46, R64
Cachexia	R64
Acute Lymphoblastic Leukemia (ALL)	C91.00, C91.01, C91.02
Multiple Myeloma	C90.00, C90.01, C90.02, C90.10
Diffuse Large B-Cell Lymphoma (DLBCL)	C83.30, C83.31, C83.32, C83.33
Other Non-Hodgkin Lymphomas (oNHL)	C82.00, C82.01, C82.02, C82.03, C82.04, C82.05, C82.06, C82.07, C82.08, C82.09, C82.10, C82.11, C82.12, C82.13, C82.14, C82.15, C82.16, C82.17, C82.18, C82.19, C82.20, C82.21, C82.22, C82.23, C82.24, C82.25, C82.26, C82.27, C82.28, C82.29, C82.30, C82.31, C82.32, C82.33, C82.34, C82.35, C82.36, C82.37, C82.38, C82.39, C82.40, C82.41, C82.42, C82.43, C82.44, C82.45, C82.46, C82.47, C82.48, C82.49, C82.50, C82.51, C82.52, C82.53, C82.54, C82.55, C82.56, C82.57, C82.58, C82.59, C82.60, C82.61, C82.62, C82.63, C82.64, C82.65, C82.66, C82.67, C82.68, C82.69, C82.80, C82.81, C82.82, C82.83, C82.84, C82.85, C82.86, C82.87, C82.88, C82.89, C82.90, C82.91, C82.92, C82.93, C82.94, C82.95, C82.96, C82.97, C82.98, C82.99, C83.00, C83.01, C83.02, C83.03, C83.04, C83.05, C83.06, C83.07, C83.08, C83.09, C83.10, C83.11, C83.12, C83.13, C83.14, C83.15, C83.16, C83.17, C83.18, C83.19, C83.34, C83.35, C83.36, C83.37, C83.38, C83.39, C83.50, C83.51, C83.52, C83.53, C83.54, C83.55, C83.56, C83.57, C83.58, C83.59, C83.70, C83.71, C83.72, C83.73, C83.74, C83.75, C83.76, C83.77, C83.78, C83.79, C83.80, C83.81, C83.82, C83.83, C83.84, C83.85, C83.86, C83.87, C83.88, C83.89, C83.90, C83.91, C83.92, C83.93, C83.94, C83.95, C83.96, C83.97, C83.98, C83.99, C84.00, C84.01, C84.02, C84.03, C84.04, C84.05, C84.06, C84.07, C84.08, C84.09, C84.10, C84.11, C84.12, C84.13, C84.14, C84.15, C84.16, C84.17, C84.18, C84.19, C84.40, C84.41, C84.42, C84.43, C84.44, C84.45, C84.46, C84.47, C84.48, C84.49, C84.60, C84.61, C84.62, C84.63
Sepsis	A021, A227, A267, A327, A400, A401, A403, A408, A409, A41, A4101, A4102, A411, A412, A413, A414, A4150, A4151, A4152, A4153, A4159, A4181, A4189, A419, A427, A5486, B377, P360, P3610, P3619, P362, P3630, P3639, P364, P365, P368, P369, R6520, R6521, T8144XA, T8144XD, T8144XS
